# Novel platform for development, training, and validation of computer-assisted detection/diagnosis software

**DOI:** 10.1007/s11548-020-02132-z

**Published:** 2020-03-09

**Authors:** Yukihiro Nomura, Soichiro Miki, Naoto Hayashi, Shouhei Hanaoka, Issei Sato, Takeharu Yoshikawa, Yoshitaka Masutani, Osamu Abe

**Affiliations:** 1 Department of Computational Diagnostic Radiology and Preventive Medicine, The University of Tokyo Hospital, 7-3-1 Hongo, Bunkyo-ku, Tokyo, 113-8655 Japan; 2 Department of Radiology, The University of Tokyo Hospital, Tokyo, Japan; 3 Department of Complexity Science and Engineering, Graduate School of Frontier Sciences, The University of Tokyo, Tokyo, Japan; 4 Center for Advanced Intelligence Project, RIKEN, Tokyo, Japan; 5 Graduate School of Information Sciences, Hiroshima City University, Hiroshima, Japan

**Keywords:** Computer-assisted detection/diagnosis (CAD), Web interface, Image database, Clinical server, Docker

## Abstract

**Purpose:**

To build a novel, open-source, purely web-based platform system to address problems in the development and clinical use of computer-assisted detection/diagnosis (CAD) software. The new platform system will replace the existing system for the development and validation of CAD software, Clinical Infrastructure for Radiologic Computation of United Solutions (CIRCUS).

**Methods:**

In our new system, the two top-level applications visible to users are the web-based image database (CIRCUS DB; database) and the Docker plug-in-based CAD execution platform (CIRCUS CS; clinical server). These applications are built on top of a shared application programming interface server, a three-dimensional image viewer component, and an image repository.

**Results:**

We successfully installed our new system into a Linux server at two clinical sites. A total of 1954 cases were registered in CIRCUS DB. We have been utilizing CIRCUS CS with four Docker-based CAD plug-ins.

**Conclusions:**

We have successfully built a new version of the CIRCUS system. Our platform was successfully implemented at two clinical sites, and we plan to publish it as an open-source software project.

## Introduction

Computer-assisted detection/diagnosis (CAD) software has been developed by many research groups [1–5], and machine learning is one of the key technologies in CAD software. The development of CAD software based on machine learning consists of several steps: (1) collection of clinical data for machine learning, (2) algorithm development and initial training using the collected data, (3) evaluation of the performance and clinical usefulness of the software, and (4) iterative algorithm refinement and retraining using the data collected in the evaluation step [6, 7].

Among these steps, the collection of clinical data with sufficient quality and quantity is often the most time-consuming. Many clinically important diseases have low prevalence rates, and labeling clinical images requires expert knowledge. Evaluation of the clinical usefulness of CAD software is also challenging. Although it is easy to evaluate the standalone performance of CAD software using a known dataset, the actual usefulness of CAD software can only be evaluated in a real clinical environment, where radiologists use CAD software and record how it affects their diagnoses on a daily basis. To use CAD software in a clinical environment, it is necessary to establish a way to continuously access an in-hospital digital imaging and communications in medicine (DICOM) network and execute CAD software before interpretation by radiologists.

To address these problems, we constructed a platform for the development and validation of CAD software. This platform was named Clinical Infrastructure for Radiologic Computation of United Solutions (CIRCUS) [6, 8], which consists of two applications that worked only on the Windows operating system. One is a Windows-based image database (CIRCUS DB; database), which stores anonymized DICOM images and allows users to define pixel-based label data via a two-dimensional (2D)-based pixel-by-pixel painting user interface. The other application is a web-based CAD processing and evaluation server (CIRCUS CS; clinical server), which continuously processes DICOM images via CAD plug-ins based on Windows executable files, shows the results on a browser, and evaluates the results based on diagnosis by radiologists (clinical feedback).

Recent advances in web technology have made it possible to process a large amount of medical data directly on browsers and display images using advanced techniques such as multiplanar reconstruction (MPR) and volume rendering (VR). These techniques are helpful in grasping the shape of a lesion and define high-quality voxel-based label data [9]. In addition, an increasing number of recent CAD algorithms based on deep learning are implemented in scripting (i.e., noncompiling) languages, including Python, and they tend to depend on different runtimes, external libraries, and graphics processing units (GPUs) [3, 4]. Thus, it is necessary to prepare and maintain various CAD algorithms in a secure and isolated manner.

On the bases of these backgrounds, we have decided to build a novel, open-source, purely web-based version of CIRCUS DB/CS, as well as a DICOM viewer component with advanced viewing techniques. To the best of our knowledge, there was no open-source software solution that met all of our requirements. The objective of this study was to build a novel platform for the development and validation of CAD software. The main improvements of the new CIRCUS system are as follows:

- The new CIRCUS system works on Linux.
- The interface is accessible via common web browsers without installing a special application.
- The volume-based viewing and painting component makes it easy to define 3D shapes of labels.
- Docker-based plug-ins can drastically reduce the cost of setting up the environment to process CAD software.

## Materials and methods

**Fig. 1:**
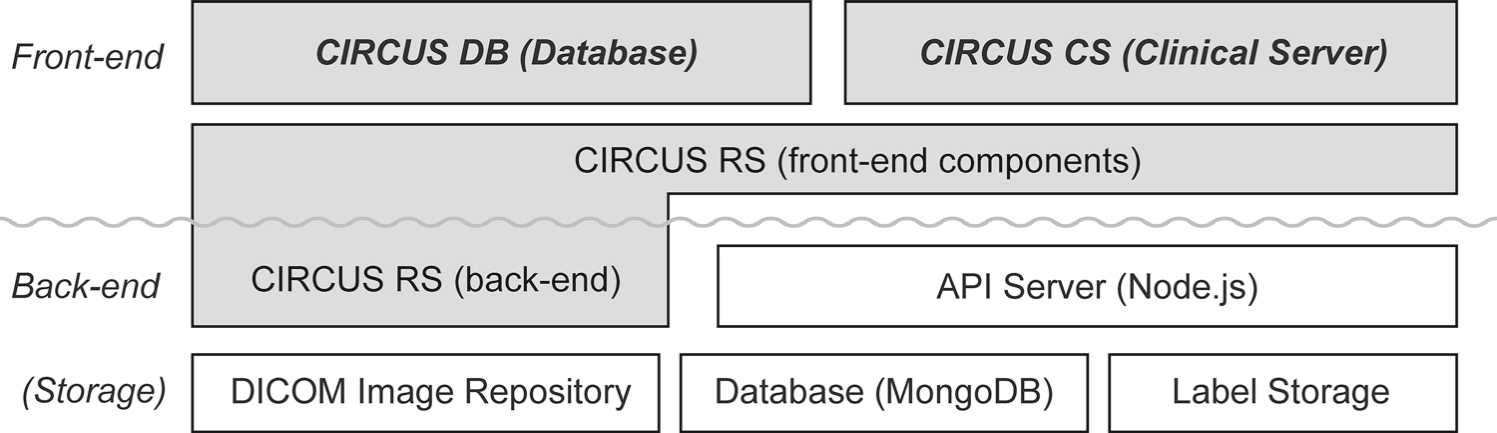
Overview of our new CIRCUS system. Two top-level applications visible to users are the new versions of CIRCUS DB and CIRCUS CS. These applications are built on top of a shared API server, CIRCUS RS, and a DICOM image repository. DB, database; CS, clinical server; RS, rendering server; API, application programming interface

Figure 1 shows an overview of the new CIRCUS system. The two top-level applications visible to users are the new versions of CIRCUS DB and CIRCUS CS. These applications are built on top of a shared application programming interface (API) server, a three-dimensional (3D) DICOM viewer component (CIRCUS RS; rendering server), and a DICOM image repository, each of which will be described in the following sections.

We chose JavaScript as the primary programming language. JavaScript runs both on the front end (browsers) and the back end (servers, via Node.js [10]), which was an important characteristic for achieving efficient image viewing, as discussed later. Other technologies we adopted included Nginx [11] (a lightweight web server used as a reverse proxy), MongoDB [12] (a NoSQL database management system that enables a flexible data structure [13, 14]), and Docker [15] (operating system virtualization software, discussed in detail later).

### CIRCUS RS: DICOM image viewer

**Fig. 2:**
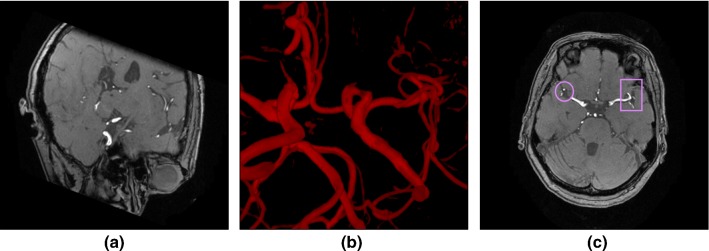
Examples of image viewing by CIRCUS RS. **a** Multiplanar reconstruction (MPR) view, **b** volume rendering (VR), **c** 2D-based region of interest (ROI) placing (ellipse or rectangle)

CIRCUS RS is a web-based DICOM viewer with support for MPR (Fig. 2a) and VR (Fig. 2b). It consists of a viewer component running on browsers and an image server library running on the server side. A key feature is a voxel-based annotation interface with which a user can define labels or view lesion candidates in a 3D space. The annotation interface also supports 2D geometrical annotation (ellipse or rectangle) (Fig. 2c). The VR code was written in OpenGL Shading Language (GLSL). The GLSL enables us to accelerate the rendering process on a dedicated GPU or central processing unit (CPU)-integrated GPU.

Since CAD software developers typically need to handle large thin-slice volume data, CIRCUS RS is optimized for this scenario. To achieve this, we implemented it so that MPR calculation can be performed both on the server side (“dynamic mode”) and the browser side (“client mode”). In the dynamic mode, the server reconstructs an MPR image for each frame, encodes it into portable network graphics (PNG), and transfers it to the browser. In the client mode, the whole volume is first transferred to the browser, and the browser performs the MPR calculation. The dynamic mode has a low frame rate because a network transfer occurs for each frame. The client mode yields a high frame rate, but it requires a large amount of memory on the browser side, and it also suffers a slow initial response. We also implemented a “hybrid mode,” where the rendering mode is seamlessly switched from the dynamic mode to the client mode after the whole voxel data have been transferred to the browser. This enables both a good initial response time and a high frame rate. The same reconstruction code written in JavaScript runs in all modes.

### API server

The API server handles all text and binary data used in CIRCUS DB and CIRCUS CS. Communication is based on JavaScript Object Notation (JSON), and a client (e.g., a browser) is authorized via an OAuth 2.0 [16] token. In the new CIRCUS system, the API layer and the user interface are clearly separated. This makes it easy for developers to integrate the CIRCUS system with various types of software, including desktop applications such as commercial DICOM viewers and reporting systems, or to write a script to analyze data managed by the CIRCUS system. Most data are stored in a MongoDB database.

### CIRCUS DB: image database

CIRCUS DB is a clinical image database application for collecting datasets used to develop and evaluate CAD software. It stores DICOM series, optionally after anonymization, and users can define labels on the stored images. The labeling part is powered by CIRCUS RS, and thus supports both 2D- and 3D-based labels.

In CIRCUS DB, clinical data are organized on the basis of a unit called a c*ase*, which has a globally unique identifier. Each case belongs to a group called a *project*. Each case can have one or more DICOM *series*, on which a user can define one or more *labels*. A case and a label can also have structured metadata called *attributes*, whose format is defined at the project level using a subset of JSON Schema [17]. Each case data have history information called *revisions*, which keep track of a list of attributes and labels data for a certain time point. It is thus possible to create a new revision based on attributes or labels registered in the past.

**Fig. 3:**
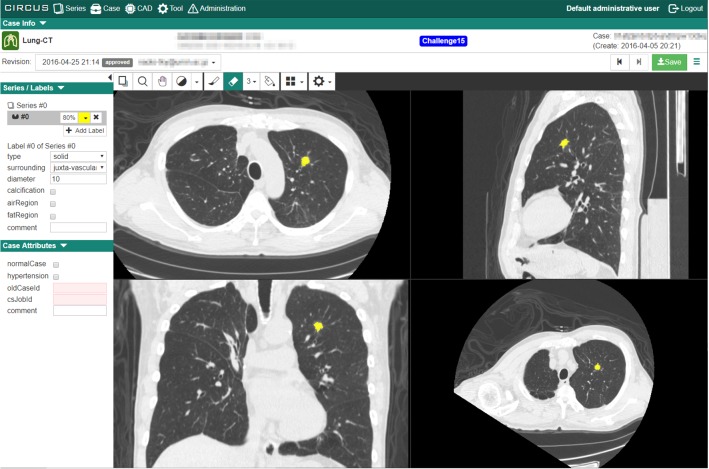
Web interface for defining gold standards and entering attributes in CIRCUS DB. The left panel has a series selector and an attribute editor. The right panel has a grid of DICOM viewer components, which includes an axial view, a sagittal view, a coronal view, and an oblique view

Figure 3 shows the web interface used to define labels and their attributes in CIRCUS DB. There is a grid of DICOM viewer components (CIRCUS RS) on the right, on which users can define labels. Users can also observe MPR images (sagittal, coronal, oblique) and define 3D voxel labels directly on them. The left panel has a series selector and an attribute editor. In this example, the case belongs to a lung nodule database project, and users assign label-based metadata (e.g., size, diameter) to each label (i.e., nodule). We took advantage of MongoDB’s flexible data structure [13, 14] to efficiently store and search these custom attributes.

As management functions, firstly, each case has history information for attributes and labels. That is, it is possible to update attributes or labels registered in the past. Second, the DICOM series belonging to each case cannot be deleted. In addition, if images of the DICOM series are added or updated after registering attributes or labels, a warning is displayed.

All the data that constitute a case can be exported and imported directly via either the API server or the web interface. Volume data can be exported as raw volume file with an Insight Toolkit (ITK) metaheader file after an anonymization process. This functionality enables us to share anonymized case data and build a larger database among multiple facilities.

### CIRCUS CS: CAD processing and evaluation server

**Fig. 4:**
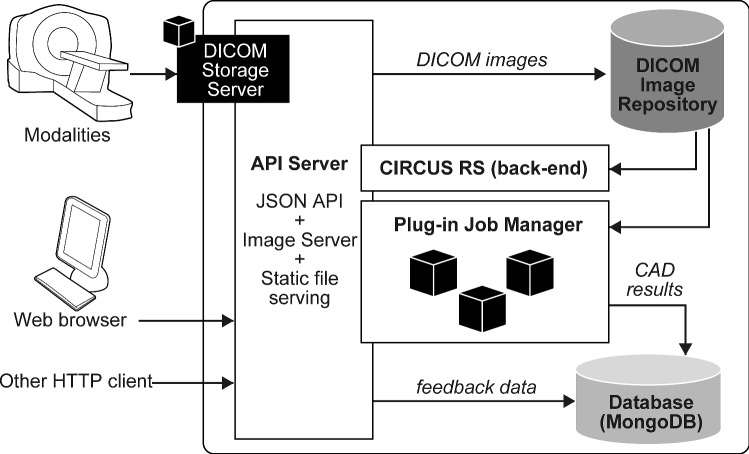
Basic configuration of CIRCUS CS. CIRCUS CS includes a DICOM storage server, a DICOM image repository, a plug-in job manager, CIRCUS RS, an API server, and a database. CAD plug-ins are provided as plug-ins of CIRCUS CS. CS, clinical server; RS, rendering server; JSON, JavaScript Object Notation; API, application programming interface

Our new version of CIRCUS CS is a CAD execution platform based on Docker plug-ins. Figure 4 shows its basic configuration. Users can request to execute a plug-in as a *job* on selected DICOM series, and then each job is sequentially processed by CIRCUS CS Job Manager. The results are either displayed as a web page or fetched via the API server.

Docker is a lightweight virtualization technology that handles an application contained in a secure and isolated package called an image. Plug-ins of the new CIRCUS CS are implemented and installed as a Docker image. The image can have the main application and its associated runtimes and libraries contained in one package, which makes it easy to manage various applications written in different programming environments. With nvidia-docker (nvidia-container-toolkit or nvidia-docker2) [18], it is also possible to execute plug-ins using a GPU.

For security reasons, we decided not to provide raw DICOM series to CAD plug-ins. Instead, in the preprocessing phase of each job, Job Manager converts DICOM series into a raw volume file with an ITK metaheader file, and extracts selected DICOM metadata into plain-text files. With this approach, plug-in developers do not need to parse DICOM files, and patient information will not be accessible to plug-ins. In addition, a running Docker-based CAD plug-in has no network connection, and has access to only a temporary directory created and mounted for each job.

**Fig. 5:**
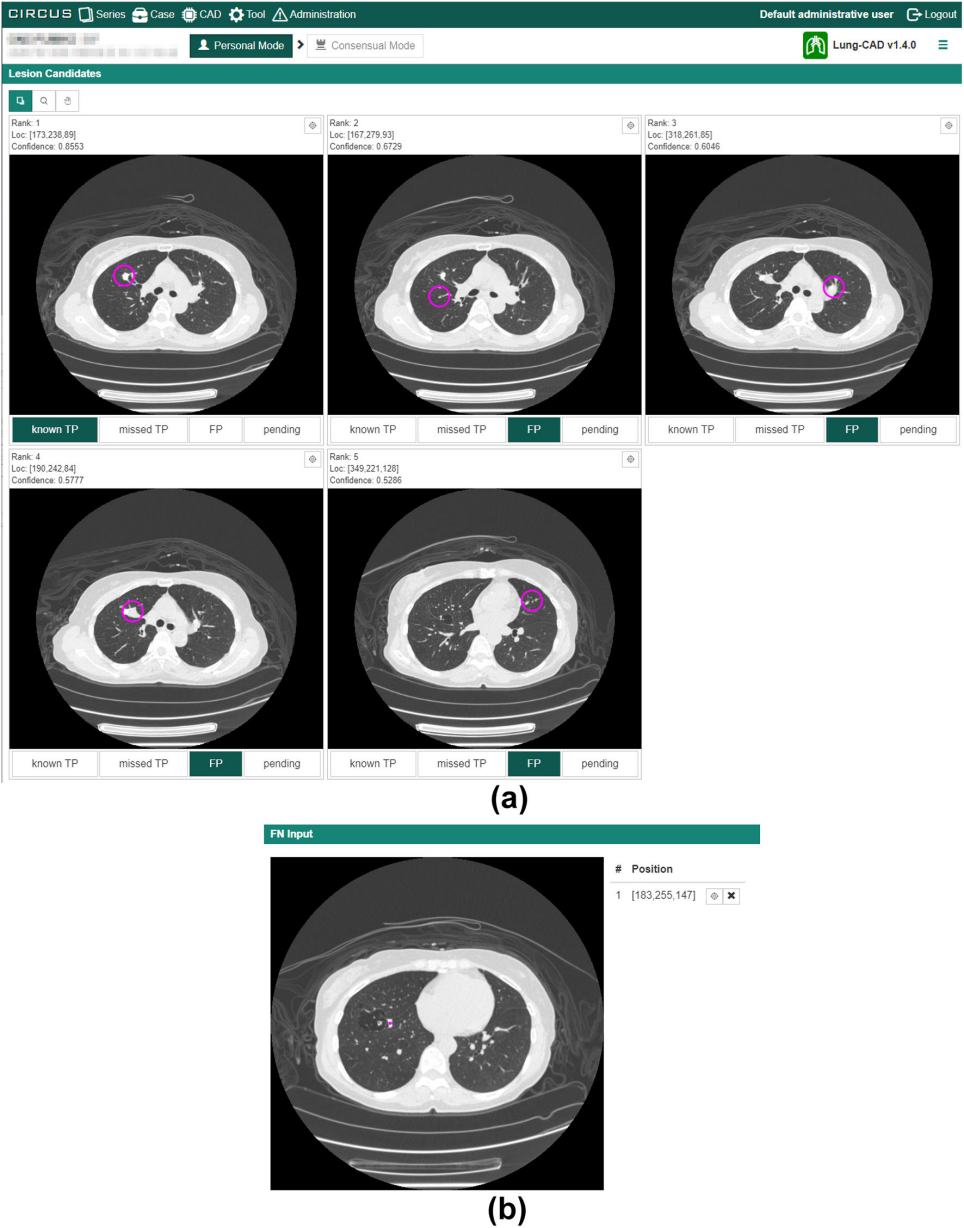
Result of lung nodule detection in chest CT images (Lung-CAD). **a** Lesion classification interface. The top five target nodules are displayed, each of which has toggle buttons to select “known TP,” “missed TP,” “sub TP,” “FP,” or “pending.” Circles indicate the locations of lesion candidates. **b** False-negative input interface. Radiologists record the location of a nodule by a mouse click if the CAD software does not display it. TP, true positive; FP, false positive

Figure 5a shows an example of the result page of a CAD job. Each lesion candidate is displayed using CIRCUS RS. For each lesion candidate, the system can collect feedback data from users, which is stored in the database and can be used for various purposes including evaluation and retraining of the CAD software. In this example, four toggle buttons are displayed (“known TP,” “missed TP,” “FP,” and “pending”; TP, true positive; FP, false positive) so that the system can record whether each lesion candidate was a correct lesion as well as whether it was useful to the user of the system. The four toggle buttons are defined as follows:

- Known TP: a true lesion detected in a radiologist’s interpretation without CAD software
- Missed TP: a true lesion overlooked in a radiologist’s interpretation without CAD software
- FP: a lesion candidate that is clearly not a true lesion
- Pending: a lesion candidate that is difficult to classify into TP or FP

The feedback collection mechanism can be configured to use other user interface elements, such as textboxes and sliders. Additionally, it is possible to collect feedback that is not tied to individual lesion candidates. In this example, an interface to tell the locations of false-negative lesions is shown (Fig. 5b).

### Image transfer and DICOM storage

We implemented three methods to import DICOM images from external sources: (1) a browser-based image uploader, (2) a command-line interface written in JavaScript, and (3) A Docker-based DICOM storage server that supports DICOM Storage Service Class Provider (SCP) protocol. Images are stored via DICOM repository, a filesystem abstraction layer that supports various storage systems. To manage access privilege, a string value called *domain* is assigned to each DICOM series.

### Building the Docker images

We created Docker-based versions of three existing CIRCUS CS plug-ins:

- Cerebral aneurysm detection in magnetic resonance (MR) angiograms based on 3D local intensity structure analysis [7] (hereafter, MRA-local)
- Lung nodule detection in chest computed tomography (CT) images [19, 20] (hereafter, Lung-CAD)
- Volumetry of visceral fat tissue (VAT) and subcutaneous fat tissue (SAT) in whole-body CT images [21] (hereafter, Fat-volumetry)

These plug-ins were initially implemented as Windows executables and have been utilized in annual whole-body general medical examinations at our hospital and other institutions with the first version of CIRCUS.

In addition, we also created a GPU-powered plug-in:

- Cerebral aneurysm detection in magnetic resonance (MR) angiograms based on convolutional neural network (CNN) [22] (hereafter, MRA-CNN)

This plug-in was implemented using Python 3.6.5 and Chainer 6.4.0 [23]. We built two Docker images of the MRA-CNN plug-in for CUDA 9.0 and CUDA 10.0.

To install our new system more easily, we also built a Docker image of the CIRCUS system that includes Node.js, Nginx, and MongoDB. In the Docker-based CIRCUS system, Docker-based CAD plug-ins are executed using the Docker outside of Docker technique [24].

### Implementation of our system

We installed our new system at two clinical sites that have been using the first version of CIRCUS. These implementations were approved by the ethical review boards of our institutions.

Site 1: The University of Tokyo Hospital.

We installed our new system into a Linux server at The University of Tokyo Hospital from scratch. The hardware and software specifications of the server are as follows: Intel Xeon Silver 2.1 GHz eight-core processor with 64 GByte RAM; NVIDIA Tesla V100 GPU; Ubuntu 16.04.4 LTS; NVIDIA Driver 390.87-0, CUDA 9.0.176; cuDNN 7.0.5; Docker 18.06.0-ce3; nvidia-docker 2.0.3 + docker18.06.0-1. For CIRCUS DB, we migrated the cases registered in the first version of the image database and registered new cases using our new web-based interface. For CIRCUS CS, we retrospectively processed the four plug-ins (MRA-local, MRA-CNN, Lung-CAD, and Fat-volumetry) for all cases of annual whole-body general medical examinations, which include chest CT, whole-body CT, and MR angiography, underwent between October 1, 2019, and October 31, 2019.

Site 2: Private cloud-based teleradiology environment.

We also installed our Docker-based system into a Linux server in a private cloud-based teleradiology environment [25]. The hardware and software specifications of the server are as follows: Intel i7-9750H 2.60 GHz six-core processor with 16 GByte RAM; NVIDIA GeForce RTX 2070 MAX-Q GPU; Ubuntu 19.10; NVIDIA Driver 440.36, CUDA 10.0.130; cuDNN 7.5.1; Docker 19.03.3; and nvidia-container-toolkit 1.0.5-1. We retrospectively processed the two plug-ins (MRA-local, MRA-CNN) for all cases of MR angiography underwent at two institutions (hospital A and clinic B) between October 1, 2019, and October 31, 2019. We also retrospectively processed the Lung-CAD plug-in for all cases of chest CT underwent at the two institutions between October 1, 2019, and October 31, 2019.

## Results

Table 1 shows the number of cases registered on our new image database at The University of Tokyo Hospital. A total of 1478 cases registered in the first version of the image database were migrated to the new image database. We registered a total of 476 cases using our new web-based interface.

**Table 1:** Number of cases registered on our new image database

	Migrated from previous database system	Newly registered
Cerebral aneurysm in MR angiograms	1050	180
Lung nodules in chest CT images	238	296
Skin lesions in PET/CT images	37	0
Visceral spaces in whole-body CT images	138	0
Spinal regions in whole-body CT images	5	0
Bone metastasis in whole-body CT images	10	0
Total	1478	476

PET, positron emission tomography

**Fig. 6:**
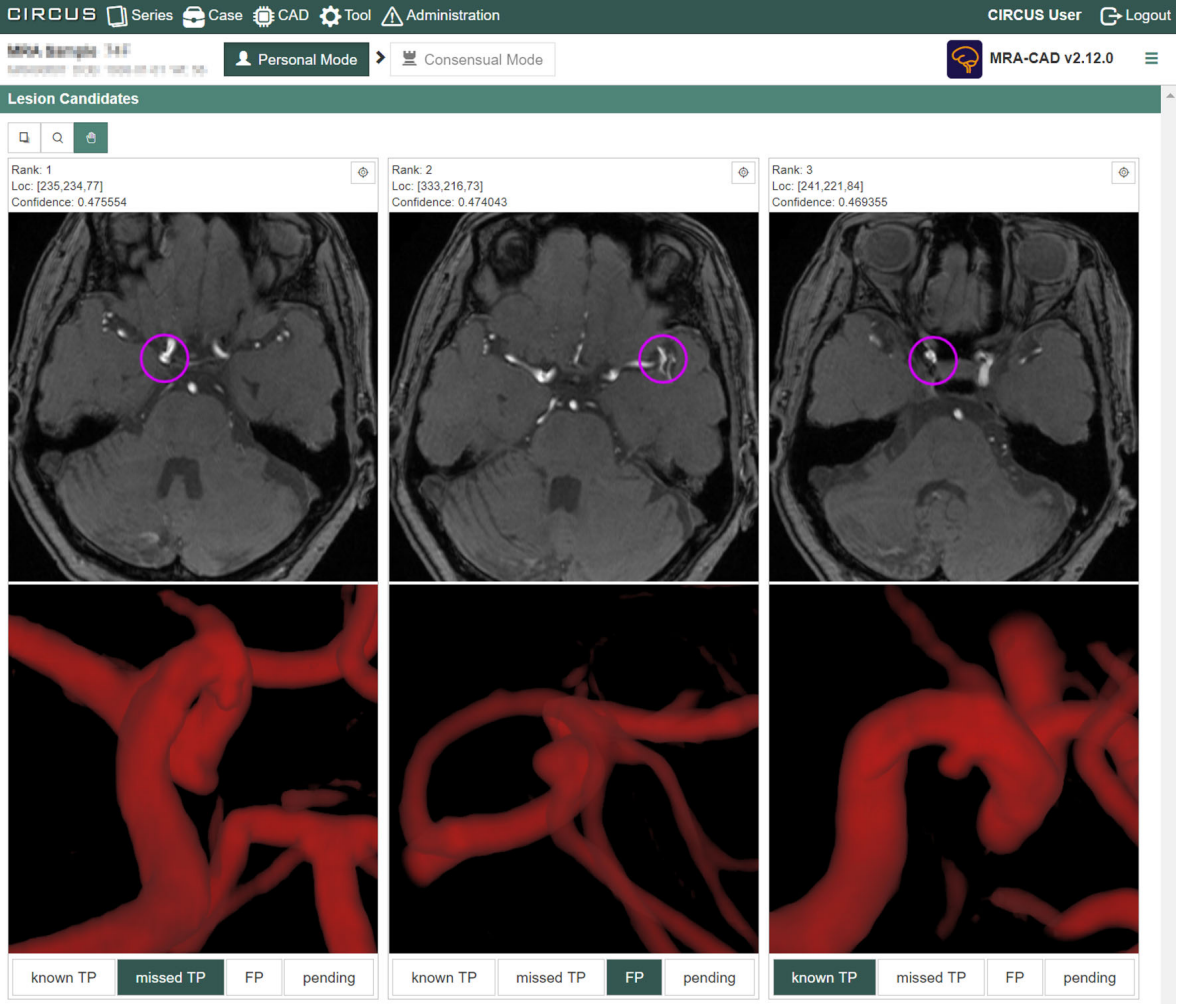
Result of cerebral aneurysm detection in MR angiograms based on 3D local intensity structure analysis (MRA-local). Not only the axial section of the lesion candidate but also partial VR images are displayed for each lesion candidate. Circles indicate the locations of lesion candidates. The result display interface of MRA-CNN is the same as that of MRA-local

**Fig. 7:**
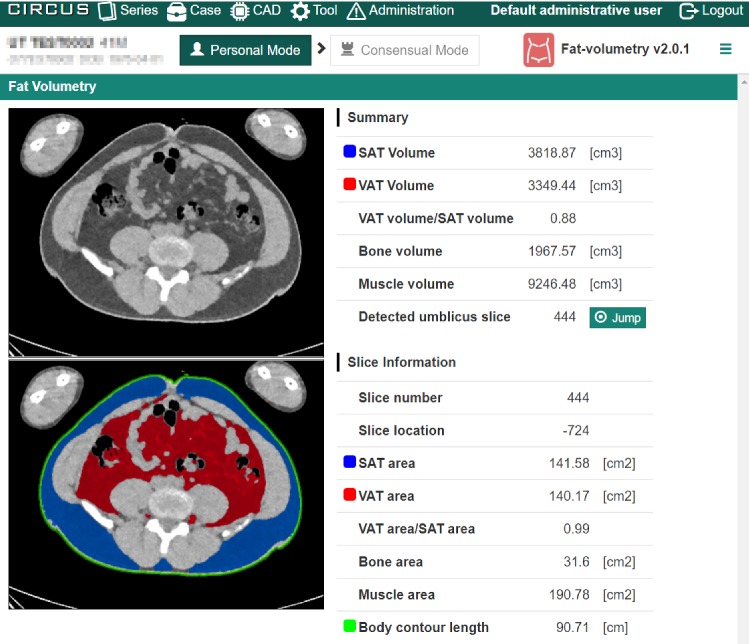
Result of volumetry of VAT and SAT in whole-body CT images (Fat-volumetry). The upper image shows a CT image in an umbilical slice, and the lower image shows extracted fat regions. The blue area represents SAT, the red area represents VAT, and the green line represents the body contour. The right tables show measurement results

Figure 5a shows a result of Lung-CAD. Figure 6 shows a result of MRA-local. In this CAD, not only the axial section of the lesion candidate but also partial VR images were displayed for each lesion candidate. The result display interface of MRA-CNN is the same as that of MRA-local. Figure 7 shows a result of Fat-volumetry. Tables 2 and 3 show the numbers of cases processed and the times required for the processing of the CAD plug-ins at the two clinical sites. There were no cases of processing failure of the CAD plug-ins.

**Table 2:** Numbers of cases processed and times required for processing of CAD plug-ins. (site: The University of Tokyo Hospital)

CAD plug-in	Number of cases	Size (*x* × *y*)	Number of images	Process time [sec]
			Median (range)	Mean ± SD (range)
MRA-local	269	632 × 768	128 (128–128)	38.7 ± 4.6 (26–57)
MRA-CNN	269	632 × 768	128 (128–128)	131.4 ± 36.6 (75–286)
Lung-CAD	262	512 × 512	302 (236–368)	92.4 ± 10.2 (66–126)
Fat-volumetry	262	512 × 512	684 (560–774)	160.5 ± 17.6 (110–225)

**Table 3:** Numbers of cases processed and times required for processing of CAD plug-ins. (site: teleradiology environment)

Institution	CAD plug-in	Number of cases	Size (*x* × *y*)	Number of images	Process time [sec]
				Median (range)	Mean ± SD (range)
Hospital A	MRA-local	34	464 × 512	224 (152–248)	39.8 ± 33.3 (14–107)
	MRA-CNN	34	464 × 512	224 (152–248)	145.8 ± 48.1 (86–295)
	Lung-CAD	149	512 × 512	321 (170–400)	73.4 ± 12.1 (39–114)
Clinic B	MRA-local	67	512 × 512	204 (200–204)	29.9 ± 3.3 (22–38)
	MRA-CNN	67	512 × 512	204 (200–204)	131.5 ± 32.7 (69–221)
	Lung-CAD	88	512 × 512	651 (306–776)	122.5 ± 17.3 (73–178)

## Discussion

We have built a novel platform for the development and validation of CAD software. The platform is readily accessible via common web browsers without installing a special application. The platform was successfully implemented at the two clinical sites, and the four CAD plug-ins were successfully processed. We plan to publish our platform as an open-source project.

The API-based system architecture enables developers to integrate CIRCUS with other medical systems. For example, in the radiology reporting system at our hospital, a button to open a web page of CAD processing result was implemented, and the result of Fat-volumetry can be exported to the reporting system.

**Fig. 8:**
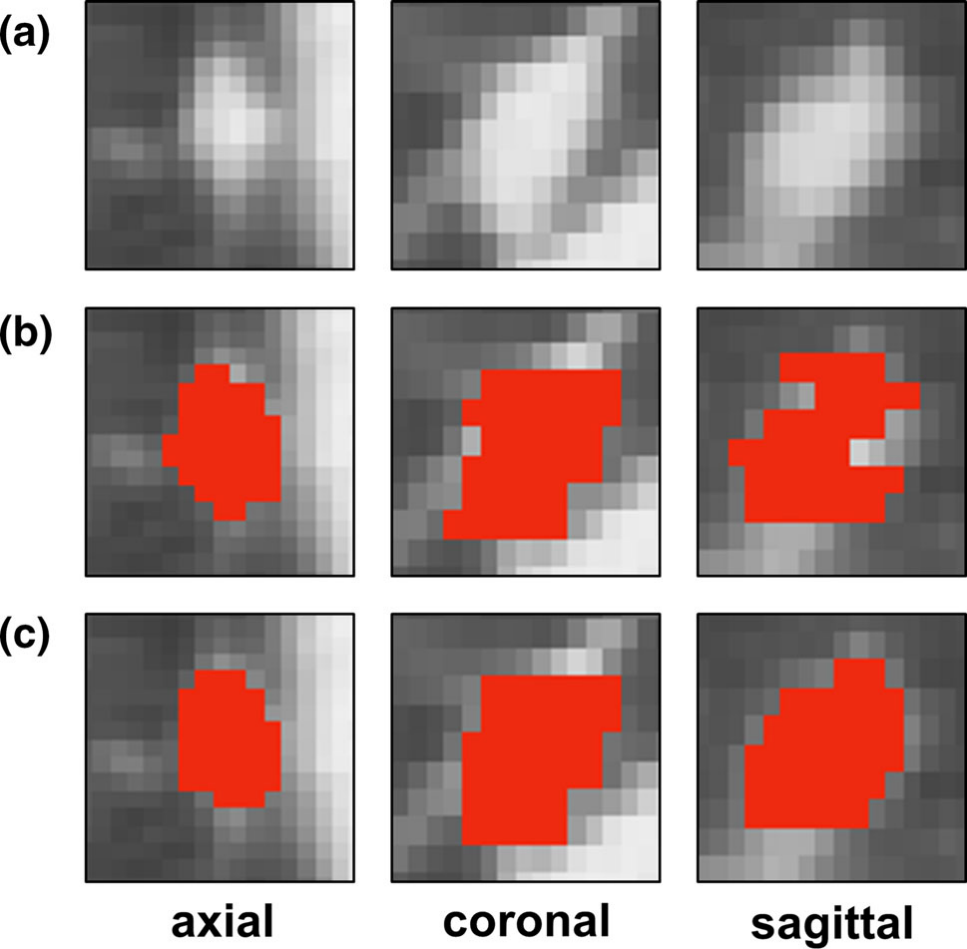
Example of voxel-based label definition (lung nodule, 9 mm). **a** Original image, **b** painted label (red area) made with our first version of CIRCUS DB (2D-based), **c** painted label defined with our new system with the aid of MPR

CIRCUS RS has the image display and annotating functions required for the entire CIRCUS system. Table 4 shows a function list of various open-source and web-based DICOM viewers including CIRCUS RS. To the best of our knowledge, there was no other web-based open-source project that satisfied all the requirements for our system. Notably, 3D voxel painting is one of the key features. Although this feature is available on several open-source desktop applications such as ITK-SNAP [9] and 3D Slicer [26], our system works without installing dedicated software on the client side. The MPR feature and the 3D voxel painting are expected to enable users to define complex 3D shapes of labels with improved precision. Although it is technically possible to construct similar data by “stacking” 2D freehand annotations, this approach is error-prone and produces unnatural jagged edges when observed in a 3D space [9] (Fig. 8).

**Table 4:** Function list of open-source and web-based DICOM viewers

Name	Version	References	MPR	Rendering	ROI	Freehand	Painting
Cornerstone	2.2.8	[27]	–	–	–	–	–
DWV	0.26.0	[28]	–	–	+	+	–
Slice:Drop	2019-07-18	[29]	+	VR	–	–	–
OHIF viewer	3.3.8	[30]	–	–	+	–	–
Papaya	Build-1449	[31]	+	SR	–	–	–
CIRCUS RS	0.24.0		+	VR	+	–	+

MPR, multiplanar reconstruction; ROI, region of interest; SR, surface rendering; VR, volume rendering; DWV, DICOM Web Viewer

We previously measured the workload of radiologists required to define 3D voxel labels by pixel-based painting using the new version of CIRCUS DB [32]. The average time required for 3D voxel labeling was 210.5 ± 119.6 s for cerebral aneurysms and 403.3 ± 277.9 s for lung nodules. The time depended on the number, size, and shape of lesions. To reduce the time required for 3D voxel labeling, we plan to develop a generalized semiautomatic segmentation function based on deep learning.

There are two web-based image database systems similar to CIRCUS DB. MD.ai Annotator [33] is a web-based tool to curate and annotate labeled datasets for machine learning training and validation. The system provides several 2D annotation methods including bounding box, freeform, polygon, and location, and allows users to collaboratively label and annotate in real time. Although there are several public projects including RSNA 2018 Machine Learning Challenge [34], at the time of writing, personal projects cannot be created. OHIF LesionTracker [30] is a web-based platform designed to facilitate quantitative assessments of tumor burden over time. The platform supports 2D annotation (bounding boxes or ellipses).

The Docker-based approach of CIRCUS CS enables researchers to manage various CAD applications more easily in clinical environments. Today, an increasing number of CAD applications are written in scripting languages, and they tend to depend on many runtimes and libraries involving thousands of files. Docker-based plug-ins can drastically reduce the cost to set up the environment to run each plug-in. We implemented the MRA-CNN as a GPU-powered plug-in. We are also developing other CAD applications using deep learning [35, 36] and plan to implement these applications.

There are two systems similar to CIRCUS CS. M5L on-demand Lung-CAD [37, 38] is a web- and cloud-based CAD system dedicated to the automatic detection of pulmonary nodules. The detection algorithm is a combination of two independent algorithms: the Channeler Ant Model (lungCAM) and the voxel-based neural approach (VBNA). Its result page has radio buttons to classify the CAD findings. EnvoyAI [39] is a cloud intelligence platform that offers multiple algorithms developed by various organizations. However, EnvoyAI is a commercialized service, and the platform is not open source. By contrast, our system is open source and can be installed in any environment, including public cloud environments such as Amazon Web Services. If clinical data are to be stored in a cloud environment, it is necessary to comply with privacy laws and guidelines in each state and country. In particular, the physical location of the cloud infrastructure is critical [40, 41]. We believe our open-source approach can help researchers, and clinicians use CAD software more easily inside their institution.

Our study has several limitations. First, the DICOM storage server does not support Query/Retrieve Service Class User (SCU), which means a user cannot search and retrieve images from external PACS sources using CIRCUS. Second, the results from CIRCUS CS plug-ins cannot be output as a DICOM structured report (SR) or a grayscale softcopy presentation state (GSPS). These are sometimes useful for integrating our system with existing reading environments, although the integration results in the loss of feedback collection mechanisms. Third, the job manager of CIRCUS CS does not support the concurrent execution of multiple jobs. To address this issue and achieve scalability, we are considering the use of a container orchestration system such as Kubernetes [42].

## Conclusion

We have successfully built a novel platform for the development and validation of CAD software, named CIRCUS. Our platform was successfully implemented at our hospital, and we plan to publish it as an open-source software project.
